# Supplementary material for: Literature Review of BARD1 as a Cancer Predisposing Gene with a Focus on Breast and Ovarian Cancers
Source: Genes (Basel). 2020 Jul 27;11(8):856. doi: 10.3390/genes11080856 (PMC7464855; doi:10.3390/genes11080856)
Supplement: Supplementary file 1 [file genes-11-00856-s001.zip › 2020.06.30_V8_BARD1_supplementary_methods_FINAL.docx]

Literature Review and Wed-Based Searches

PubMed (www.pubmed.ncbi.nlm.nih.gov) was searched for articles between May 1, 2020–June 30, 2020 using the following terms: *BARD1*, hereditary cancer, *in vitro* assay, functional characterization, biological function, sequencing, panel testing and next-generation sequencing. Articles containing studies addressing the following subjects were retrieved: i) *BARD1* sequencing in hereditary cancer cases; ii) *in vitro* or *in cellulo* assays performed to functionally characterize *BARD1* variants; and iii) association of *BARD1* with cancer risk. Variants that appear in the **Supplementary Table** were identified in cancer cases from this literature review.

An ad hoc web-based search was performed to identify North American based commercial genetic testing companies that include *BARD1* as a part of gene panel testing.

Bioinformatic Tools Used for Evaluating Variants

- Variant effect predictor (VEP; grch37.ensembl.org/Homo_sapiens/Tools/VEP?db=core) was used to annotate variants with the following *in silico* tools:
  - Combined Annotation Dependent Depletion (CADD) v1.4 [2]
  - Eigen v1.1 [3]
  - Meta Logistic Regression (MetaLR) [4]
  - Meta Support Vector Machine (MetaSVM) [4]
  - Protein Variant Effect Analyzer (PROVEAN) v1.1 [5]
  - Rare Exome Variant Ensemble Learner (REVEL) [6]
  - Variant Effect Scoring Tool (VEST) v4.0 [7]
  - dbscSNV [8]
  - Site-specific Phylogenetic analysis (SiPhy) [9]
  - Phylogenetic P-values (PhyloP) 100 way in vertebrates [10]
  - Genomic Evolutionary Rate Profiling (GERP++) [11]
- The following *in silico* tools were chosen as the algorithms were shown to have high performance characteristics across different data sets [12].
- Human Splicing Finder (Version 3.1; www.genomnis.com/access-hsf) was used to annotate variants for potential impact on splicing.
- Varsome (www.varsome.com) [14] was used to determine the American College of Medical Genetics and Genomics (ACMG) classification (pathogenic, likely pathogenic, uncertain significance, likely benign, benign).
- ClinVar database (www.ncbi.nlm.nih.gov/clinvar/) [15] was used to determine the clinical interpretation of identified variants and was last accessed on June 22, 2020.
- National Center for Biotechnology Information (NCBI) Protein (www.ncbi.nlm.nih.gov/protein/) was used to determine the protein domains of BARD1 (CAE48237.1).
- BARD1 exon sizes were determined using the University of California Santa Cruz (UCSC; www.genome.ucsc.edu) Genome Browser [16].
- All frameshift, nonsense and canonical splice site (+/-5 nucleotides from the exon) variants were considered to result in loss of function.
- All missense variants were considered potentially pathogenic if variants were predicted to be damaging in at least five out of seven *in silico* tools (CADD, Eigen, MetaLR, MetaSVM, PROVEAN, REVEL and VEST) and conserved in all three *in silico* conservation tools (SiPhy, PhyloP and GERP++).
- Synonymous variants were assumed to be benign as they did not create cryptic splice sites based on *in silico* tools.

Informatic Tools Used to Evaluate Allele Frequency and Prevalence

- The Interactive Prevalence Tables From Multi-Gene Panel Testing: A collaboration between investigators from Mayo Clinic and Ambry Genetics® [1] (www.ambrygen.com/clinician/resources/prevalence-tool) was investigated for prevalence of pathogenic variants in *BARD1* from a clinical testing company.
- Genome Aggregation Database (gnomAD v2.1.1 [non-cancer]; www.gnomad.broadinstitute.org) [13] was used to determine the allele frequency of *BARD1* variants in non-cancer individuals across populations.

**References**

1. Hart, S.N.; Polley, E.C.; Yussuf, A.; Yadav, S.; Goldgar, D.E.; Hu, C.; LaDuca, H.; Smith, L.P.; Fujimoto, J.; Li, S.; et al. Mutation prevalence tables for hereditary cancer derived from multi‐gene panel testing. *Hum. Mutat.* **2020**, *92*, 220–225, doi:10.1002/humu.24053.

2. Rentzsch, P.; Witten, D.; Cooper, G.M.; Shendure, J.; Kircher, M. CADD: predicting the deleteriousness of variants throughout the human genome. *Nucleic Acids Res.* **2019**, *47*, D886–D894, doi:10.1093/nar/gky1016.

3. Ionita-Laza, I.; McCallum, K.; Xu, B.; Buxbaum, J.D. A spectral approach integrating functional genomic annotations for coding and noncoding variants. *Nat. Genet.* **2016**, *48*, 214–220, doi:10.1038/ng.3477.

4. Dong, C.; Wei, P.; Jian, X.; Gibbs, R.; Boerwinkle, E.; Wang, K.; Liu, X. Comparison and integration of deleteriousness prediction methods for nonsynonymous SNVs in whole exome sequencing studies. *Hum. Mol. Genet.* **2015**, *24*, 2125–2137, doi:10.1093/hmg/ddu733.

5. Choi, Y.; Sims, G.E.; Murphy, S.; Miller, J.R.; Chan, A.P. Predicting the Functional Effect of Amino Acid Substitutions and Indels. *PLoS One* **2012**, *7*, e46688, doi:10.1371/journal.pone.0046688.

6. Ioannidis, N.M.; Rothstein, J.H.; Pejaver, V.; Middha, S.; McDonnell, S.K.; Baheti, S.; Musolf, A.; Li, Q.; Holzinger, E.; Karyadi, D.; et al. REVEL: An Ensemble Method for Predicting the Pathogenicity of Rare Missense Variants. *Am. J. Hum. Genet.* **2016**, *99*, 877–885, doi:10.1016/j.ajhg.2016.08.016.

7. Douville, C.; Masica, D.L.; Stenson, P.D.; Cooper, D.N.; Gygax, D.M.; Kim, R.; Ryan, M.; Karchin, R. Assessing the Pathogenicity of Insertion and Deletion Variants with the Variant Effect Scoring Tool (VEST-Indel). *Hum. Mutat.* **2016**, *37*, 28–35, doi:10.1002/humu.22911.

8. Jian, X.; Boerwinkle, E.; Liu, X. In silico prediction of splice-altering single nucleotide variants in the human genome. *Nucleic Acids Res.* **2014**, *42*, 13534–13544, doi:10.1093/nar/gku1206.

9. Garber, M.; Guttman, M.; Clamp, M.; Zody, M.C.; Friedman, N.; Xie, X. Identifying novel constrained elements by exploiting biased substitution patterns. *Bioinformatics* **2009**, *25*, i54–i62, doi:10.1093/bioinformatics/btp190.

10. Pollard, K.S.; Hubisz, M.J.; Rosenbloom, K.R.; Siepel, A. Detection of nonneutral substitution rates on mammalian phylogenies. *Genome Res.* **2010**, *20*, 110–121, doi:10.1101/gr.097857.109.

11. Davydov, E. V; Goode, D.L.; Sirota, M.; Cooper, G.M.; Sidow, A. Identifying a high fraction of the human genome to be under selective constraint using GERP ++. *PLoS Comput. Biol.* **2010**, *6*, e1001025, doi:10.1371/journal.pcbi.1001025.

12. Ghosh, R.; Oak, N.; Plon, S.E. Evaluation of in silico algorithms for use with ACMG/AMP clinical variant interpretation guidelines. *Genome Biol.* **2017**, *18*, doi:10.1186/s13059-017-1353-5.

13. Karczewski, K.J.; Francioli, L.C.; Tiao, G.; Cummings, B.B.; Alföldi, J.; Wang, Q.; Collins, R.L.; Laricchia, K.M.; Ganna, A.; Birnbaum, D.P.; et al. The mutational constraint spectrum quantified from variation in 141,456 humans. *Nature* **2020**, *581*, 434–443, doi:10.1101/531210.

14. Kopanos, C.; Tsiolkas, V.; Kouris, A.; Chapple, C.E.; Albarca Aguilera, M.; Meyer, R.; Massouras, A. VarSome: the human genomic variant search engine. *Bioinformatics* **2018**, *35*, 1978–1980, doi:10.1093/bioinformatics/bty897.

15. Landrum, M.J.; Lee, J.M.; Benson, M.; Brown, G.R.; Chao, C.; Chitipiralla, S.; Gu, B.; Hart, J.; Hoffman, D.; Jang, W.; et al. ClinVar: Improving access to variant interpretations and supporting evidence. *Nucleic Acids Res.* **2018**, *46*, 1062–1067, doi:10.1093/nar/gkx1153.

16. Kent, W.J.; Sugnet, C.W.; Furey, T.S.; Roskin, K.M.; Pringle, T.H.; Zahler, A.M.; Haussler, a. D. The Human Genome Browser at UCSC. *Genome Res.* **2002**, *12*, 996–1006, doi:10.1101/gr.229102.
